# USP11 augments TGFβ signalling by deubiquitylating ALK5

**DOI:** 10.1098/rsob.120063

**Published:** 2012-06

**Authors:** Mazin A. Al-Salihi, Lina Herhaus, Thomas Macartney, Gopal P. Sapkota

**Affiliations:** Medical Research Council – Protein Phosphorylation Unit, College of Life Sciences, University of Dundee, Dow St., Dundee DD1 5EH, UK

**Keywords:** USP11, USP15, TGFβ, ALK5, ubiquitin, cancer

## Abstract

The TGFβ receptors signal through phosphorylation and nuclear translocation of SMAD2/3. SMAD7, a transcriptional target of TGFβ signals, negatively regulates the TGFβ pathway by recruiting E3 ubiquitin ligases and targeting TGFβ receptors for ubiquitin-mediated degradation. In this report, we identify a deubiquitylating enzyme USP11 as an interactor of SMAD7. USP11 enhances TGFβ signalling and can override the negative effects of SMAD7. USP11 interacts with and deubiquitylates the type I TGFβ receptor (ALK5), resulting in enhanced TGFβ-induced gene transcription. The deubiquitylase activity of USP11 is required to enhance TGFβ-induced gene transcription. *RNAi*-mediated depletion of USP11 results in inhibition of TGFβ-induced SMAD2/3 phosphorylation and TGFβ-mediated transcriptional responses. Central to TGFβ pathway signalling in early embryogenesis and carcinogenesis is TGFβ-induced epithelial to mesenchymal transition. USP11 depletion results in inhibition of TGFβ-induced epithelial to mesenchymal transition.

## 2. Introduction

The signalling pathways downstream of the transforming growth factor β (TGFβ) family of receptors play critical roles in regulating cellular proliferation, apoptosis, differentiation and migration [1–3]. TGFβ pathway aberrations have been reported in a wide range of musculoskeletal, cardiovascular, reproductive and neurological pathologies both acquired and developmental [4]. Furthermore, malfunction of the TGFβ pathway is associated with cancer and metastasis, and the loss of TGFβ cytostatic responsiveness is a characteristic of many cancers [2,5]. Therefore, understanding TGFβ pathway regulation may present new opportunities for the development of novel target-specific therapeutic interventions. While multiple articles have been published on the subject, many gaps remain in our knowledge of TGFβ pathway regulation, especially after signalling has been initiated.

Signalling is initiated when TGFβ ligands bind to their transmembrane serine/threonine kinase cognate receptors. Ligand binding induces specific pairing of type I (ALK1-7) and type II (ACVR-IIA, ACVR-IIB, BMPR-II, AMHR-II and TGFβR-II) receptors in a quaternary complex. Type II receptors phosphorylate and activate the type I receptors. SMAD proteins are the intracellular signal transducers of activated receptor complexes and are divided into three groups: receptor-regulated (R-) SMADs (1–3, 5 and 8), the co-SMAD (4) and the inhibitory (I-) SMADs (6 and 7). Once activated, type I receptors phosphorylate different R-SMADs at their C-terminal SXS motif depending on the receptor pairing and the ligand. This induces R-SMAD complex formation with SMAD4 and translocation to the nucleus, where along with other cofactors they regulate transcription of more than 500 target genes. The I-SMADs are transcriptionally induced by TGFβ and bone morphogenic protein (BMP), creating a negative feedback by targeting the receptors for ubiquitin-mediated degradation and competing with R-SMADs for association with the type I receptors [6,7]. The TGFβ subfamily of receptors signal through SMADs 2 and 3 and are inhibited by SMAD7, while the BMP subfamily signal through SMADs 1, 5 and 8 and are inhibited by both SMADs 6 and 7. However, some crosstalk between the two pathways has been reported [1,8].

Regulation of the TGFβ pathway can occur at multiple levels and by various molecular mechanisms. One of the key modes of regulation is by reversible ubiquitylation of the protein components driving the TGFβ pathway. Ubiquitin is a member of a conserved family of eukaryotic proteins sharing the ubiquitin fold structure. Attached through an isopeptide bond to lysine residues of target proteins, they are used as modifiers of localization, stability and activity. Additional ubiquitins can be attached to one of the several lysine residues on the protein-bound ubiquitin, creating polyubiquitin chains. Depending on the type of polyubiquitin chains formed, different fates await the polyubiquitylated protein. Although several chain types exist, not all have been attributed a function. Of the commonly studied, K48 chains are known to signal degradation, whereas K63 chains play a role in signalling as well as protein trafficking and endocytosis [9,10].

Ubiquitin attachment is achieved through a three-step process using ubiquitin activating (E1) and conjugating (E2) enzymes, as well as a wide array of (E3) ligases [9]. Specific to the TGFβ pathway, the E3 ubiquitin ligases SMURF1 and NEDD4L attenuate TGFβ signalling by ubiquitylating SMAD1 and SMAD2/3, respectively [11–15]. SMAD4 is regulated by reversible ubiquitylation [16]. SMAD7 recruits SMURF1/2, WWP1 and NEDD4L targeting the type I receptors for ubiquitin-mediated degradation [7,17,18]. SMAD7 itself is a target for ubiquitylation by the E3 ligase ARKADIA [19]. A further layer of control is exerted on the pathway by editing or removing the ubiquitin chains from targeted pathway members, therefore changing their fate and localization. While the regulation of the TGFβ pathway by ubiquitylation has been extensively investigated and reported, deubiquitylation has not [11]. Consequently, there have been very few deubiquitylating enzymes (DUBs) reported to act on the TGFβ pathway [16,20–22].

There are at least 79 DUBs encoded in the human genome that are responsible for editing and removing ubiquitin chains by cleaving the isopeptide bond [23]. In this study, we introduce USP11 as a DUB capable of regulating the TGFβ pathway. USP11 and 56 other USP proteins share the USP domain; this contains the two or three amino acid residues forming the catalytic diad or triad required to cleave ubiquitin chains. They diverge structurally with various regulatory, ubiquitin binding and protein binding domains directing them to different targets. USP11 has been described to be involved in other pathways. It has been shown to associate with: RanBPM in the nucleation of microtubules, IκBα in the TNFα pathway, BRCA2 in DNA repair and HPV-16E7 enhancing HPV virus replication in relation to cervical cancer while inhibiting influenza virus replication. These functions among others have been reported to be both dependent and independent of its DUB activity [24–30]. Here, we report USP11 as a TGFβ pathway DUB capable of modulating TGFβ-induced signalling and downstream cellular functions.

## 3. Results

### 3.1. Identification of USP11 as an interactor of GFP-SMAD7

**Figure 1.**
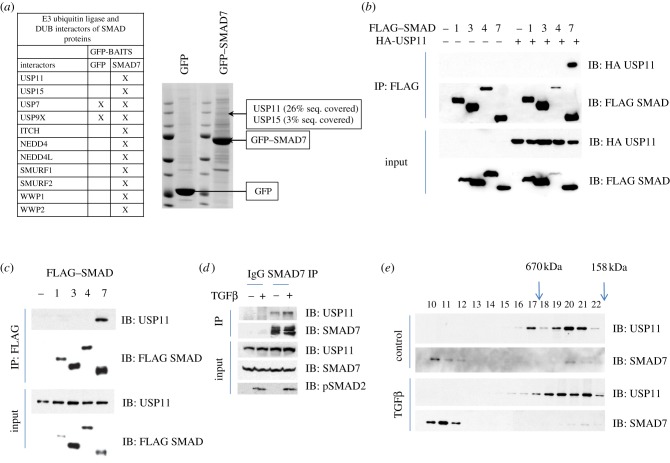
Identification and characterization of USP11 as an interactor of SMAD7. (*a*) Representative Coomassie-stained gels showing anti-GFP IPs from HEK293 extracts expressing GFP-alone or GFP-SMAD7. The interacting proteins were excised as 2 mm gel pieces, digested with trypsin and identified by mass spectrometry. The gel piece from which USP11 was identified is indicated. A summary table of various Smad-interacting E3 ubiquitin ligases and DUBs identified by mass spectrometry is included. The sequence coverage of USP11 and USP15 in GFP-SMAD7 IPs is indicated. (*b*) HEK293 cells were co-transfected transiently with HA-USP11 and FLAG–SMADs. FLAG IPs and lysate inputs were immunoblotted with FLAG and HA antibodies as indicated. (*c*) HEK293 cells were transiently transfected with FLAG–SMADs only. FLAG IPs and lysate inputs were immunoblotted with FLAG and endogenous USP11 antibodies. (*d*) Lysates from HEK293 cells treated with vehicle or TGFβ (50 pM 45 min) were immunoprecipitated using pre-immune IgG or a SMAD7 antibody covalently bound to Dynabeads (Invitrogen). IPs and lysate inputs were immunoblotted with endogenous USP11, SMAD7 and phospho-SMAD2 antibodies. (*e*) Extracts from HaCaT cells starved for 4 h and stimulated with or without 50 pM TGFβ for 1 h were separated by size-exclusion gel chromatography. The collected fractions were immunoblotted with anti-USP11 and anti-SMAD7 antibodies.

In order to uncover molecular mechanisms by which SMAD7 regulates the TGFβ pathway, we undertook a proteomic approach to identify novel interactors of SMAD7. We stably integrated a single copy of green fluorescent protein-tagged SMAD7 into HEK293 cells. From these cells, GFP-immunoprecipitates (IPs) were resolved by SDS–PAGE and the interacting proteins were excised, digested with trypsin and identified by mass spectrometry. The E3 ubiquitin ligases ITCH, NEDD4, NEDD4L, SMURF1/2 and WWP1/2, all members of the C2-WW-HECT family [31], were identified as selective SMAD7 interactors (figure 1*a*). Of these, SMURF1/2, WWP1 and NEDD4L have previously been reported to interact with SMAD7 and modulate the TGFβ pathway [7,17,18]. While the regulation of TGFβ signalling by SMAD7-associated E3 ubiquitin ligases has been extensively investigated and reported, we were drawn to the novel DUB interactors of SMAD7. USP11 and USP15 were identified as selective interactors of GFP-SMAD7 in three separate experiments. While USP11 coverage and intensity indicated a robust interaction, USP15 was less prominent (figure 1*a*). USP11 and USP15 did not feature as interactors of GFP-tagged SMADs1–5 in similar proteomic assays. USP7 and USP9X, the latter a reported deubiquitylase for SMAD4 [16], were also identified in the screen. However, both featured in the control GFP IPs indicating a non-specific interaction (figure 1*a*).

### 3.2. USP11 but not USP15 binds specifically to SMAD7

To confirm the specificity of the USP11–SMAD7 interaction, SMADs carrying an N-terminal FLAG tag were transiently transfected into HEK293 cells with or without the N-terminal HA-tagged USP11. We found that USP11 interacted more robustly with SMAD7 compared with any of the other SMAD proteins (figure 1*b*). FLAG–SMAD7 was also capable of immunoprecipitating endogenous USP11 (figure 1*c*). We also assessed the effect of TGFβ (50 pM, 45 min) on the ability of transfected FLAG–SMAD7 to immunoprecipitate endogenous USP11. TGFβ stimulation did not alter the SMAD7–USP11 interactions (see the electronic supplementary material, figure S1). Furthermore, we performed an endogenous SMAD7 immunoprecipitation and were able to detect endogenous USP11 in the SMAD7 IPs with or without TGFβ treatment (figure 1*d*). Despite reports that USP15 interacts with SMADs 2 and 3 [21,32], we failed to detect USP15 interacting with any of the SMAD proteins (see the electronic supplementary material, figure S2). We performed size-exclusion chromatography on HaCaT cell extracts in order to detect potential endogenous USP11 and SMAD7 complex formation. USP11 and SMAD7 eluted in molecular fractions much higher than their monomeric weights. They co-elute in the same high-molecular-weight fractions (fractions 20–21; figure 1*e*), indicating possible complex formation. However, both USP11 and SMAD7 also elute in non-overlapping high-molecular-weight fractions, implying that they probably also exist in unique complexes with other proteins. TGFβ treatment (50 pM, 45 min) did not alter the elution profile significantly (figure 1*e*).

USP11–SMAD7 interaction and potential complex formation raised two distinct possibilities for USP11 targets within the TGFβ pathway. One, we hypothesized that USP11 binds and deubiquitylates SMAD7, thereby inhibiting the TGFβ pathway. Two, we hypothesized that SMAD7 could direct USP11 DUB activity to other pathway proteins it interacts with, such as the type I TGFβ receptors, thereby enhancing pathway signalling.

### 3.3. USP11 enhances TGFβ pathway signalling

**Figure 2.**
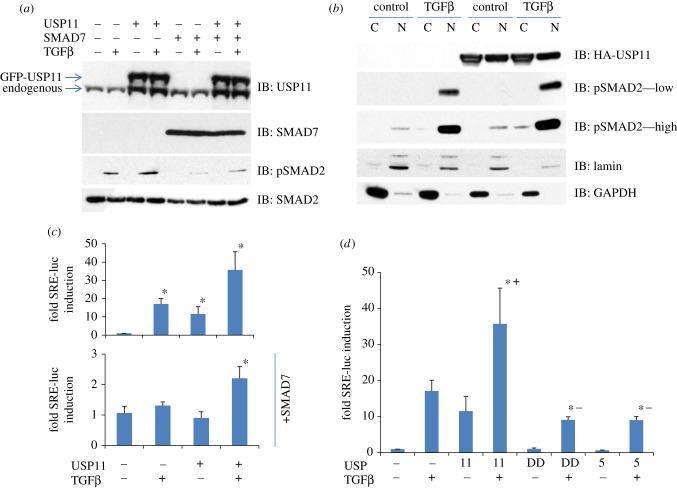
USP11 enhances TGFβ pathway signalling. (*a*) HEK293 cells stably expressing GFP or GFP-USP11 were transfected with HA empty vector or HA-SMAD7, starved for 4 h and stimulated with 50 pM TGFβ for 1 h prior to lysis. Extracts were resolved by SDS–PAGE and immunoblotted with antibodies against GFP-USP11, HA-SMAD7, endogenous phospho-SMAD2 and SMAD2. (*b*) HEK293 cells transiently transfected with or without HA-USP11 were starved for 4 h and stimulated with 50 pM TGFβ for 1 h prior to separation into cytoplasmic and nuclear fractions. The fractions were resolved by SDS–PAGE and immunoblotted with antibodies against HA-USP11, lamin, GAPDH, endogenous phospho-SMAD2 and SMAD2. All immunoblots are representative of at least three biological replicates. (*c*) TGFβ transcriptional reporter activity (using a SMAD responsive element (SRE) luciferase reporter assay) normalized to renilla-luciferase in HEK293 cells transiently transfected with SRE-luciferase, renilla-luciferase, HA-USP11, FLAG–SMAD7 and stimulated for 6 h with or without 50 pM TGFβ, as indicated. Results are average of five biological replicates. Asterisk denotes statistical significance over vector transfected and unstimulated cells. (*d*) TGFβ transcriptional reporter activity (using an SRE luciferase reporter assay) normalized to renilla-luciferase in HEK293 cells transiently transfected with SRE-luciferase, renilla-luciferase, HA-USP11, HA-C318S USP11 (DD), HA-USP5 and stimulated for 6 h with or without 50 pM TGFβ, as indicated. Results are average of three biological replicates. Asterisk denotes statistical significance over vector transfected and unstimulated cells. Plus symbols denote positive divergence, minus symbols denote negative divergence.

In order to explore the impact of USP11 on the TGFβ pathway, we used HEK293 cells stably expressing GFP or GFP-USP11 (two to threefold over endogenous USP11) with or without SMAD7 co-expression. Cells were starved for 4 h and stimulated with 50 pM TGFβ for 1 h. Cell lysates were resolved by SDS–PAGE. TGFβ-induced phospho-SMAD2 levels were slightly enhanced in cells expressing GFP-USP11 compared with the control cells. This suggested that SMAD7 is unlikely to be a substrate for USP11. As expected, SMAD7 expression resulted in significant inhibition of TGFβ-induced phosphorylation of SMAD2, which was partially rescued by USP11 (figure 2*a*).

TGFβ-induced transcriptional responses require phospho-SMAD2/3 translocate to the nucleus [33,34]. We therefore fractionated transiently transfected HEK293 cells into nuclear and cytoplasmic fractions. We found increased phospho-SMAD2 levels within the nuclear fractions in response to TGFβ and this was further enhanced by USP11 over-expression (figure 2*b*). The TGFβ-induced phospho-SMAD2 levels in the cytoplasmic fractions also increased with USP11 over-expression. Additionally, nuclear phospho-SMAD2 was enhanced by USP11 over-expression even in the absence of TGFβ stimulation (figure 2*b*). To confirm the transcriptional effect of over-expressed USP11, we transfected cells with a SMAD3-dependent TGFβ-responsive luciferase construct [35,36]. Consistent with the enhanced phospho-SMAD2 levels, USP11 significantly enhanced the TGFβ-induced reporter activity. Furthermore, USP11 was able to partially rescue the over-riding inhibitory effect of SMAD7 over-expression on TGFβ-induced transcriptional reporter activity (figure 2*c*).

We repeated the TGFβ-responsive transcriptional reporter assay with a catalytically inactive mutant of USP11 (C318S) [25]. While wild-type (wt) USP11 significantly enhanced TGFβ-induced transcriptional reporter activity, the catalytically inactive mutant USP11 (C318S) had no effect (see figure 2*d* and the electronic supplementary material, figure S3). This indicates that USP11 DUB activity is required to exert its effect on the TGFβ pathway. To determine the specificity of USP11 on TGFβ-induced transcriptional reporter activity, we tested USP5 in the same assay. While being a member of the USP family of deubiquitylases (DUBs), it did not appear in SMAD7 IPs in the proteomic screens. We found USP5 had no effect on TGFβ-induced reporter activity (figure 2*d*), implying selective effects of USP11 on the TGFβ pathway.

### 3.4. USP11 knockdown inhibits TGFβ pathway signalling

**Figure 3.**
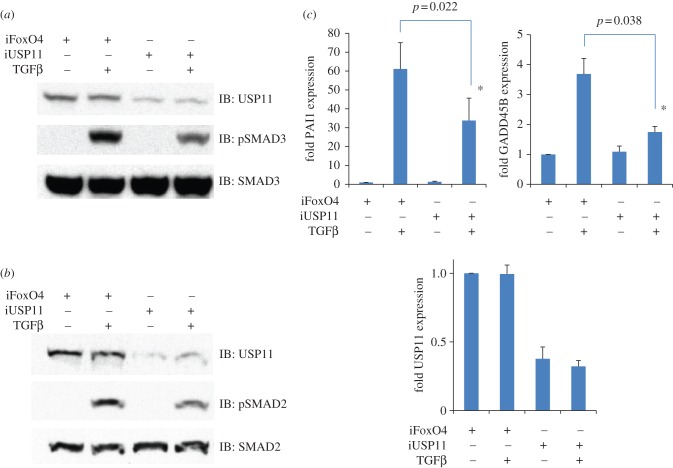
*RNAi* depletion of USP11 inhibits TGFβ pathway signalling. (*a*) HEK293 cells were transiently transfected with *siRNA* targeting FoxO4 as control or USP11, starved for 4 h and stimulated with 50 pM TGFβ for 1 h prior to lysis. Extracts were resolved by SDS–PAGE and immunoblotted with antibodies against endogenous USP11, phospho-SMAD3 and SMAD3. (*b*) As in A except that HEK293 cells were transiently transfected with *esiRNA* targeting FoxO4 as control or USP11 and immunoblots against phospho-SMAD2 and SMAD2 were performed. Immunoblots are representative of two biological replicates each, using two sets of *RNAi*. (*c*) HEK293 cells were transiently transfected with *siRNA* targeting FoxO4 as control or USP11, starved overnight and stimulated for 4 h with 50 pM TGFβ. The expression of TGFβ-target genes PAI1 and GADD45B as well as USP11 knockdown were assessed by semiquantitative RT-PCR. Results are average of three biological replicates. Asterisk denotes statistical significance.

Next, we investigated the impact of *RNAi*-mediated depletion of USP11 on TGFβ signalling. Two distinct pools of siRNAs targeting USP11 yielded a moderate reduction (60–80%) in endogenous USP11 expression, while a control siRNA targeting FoxO4 [37] did not (figure 3). Depletion of USP11 by these *RNAi* target sequences resulted in a reduction in levels of TGFβ-induced phospho-SMAD2 and 3 without affecting total SMAD2/3 levels (figure 3*a*,*b*). Consistent with these observations, *RNAi*-mediated depletion of USP11 resulted in the reduced expression of TGFβ-target genes PAI-1, and GADD45B (figure 3*c*). We also confirmed that USP11 *RNAi* did not target USP15 and vice versa, confirming that the observed effects of USP11 on the TGFβ pathway are likely to be due to USP11 (see the electronic supplementary material, figure S4).

The preceding results clearly show that USP11 affects SMAD2/3 phosphorylation; therefore, it would appear that USP11 modulates the pathway upstream of SMAD2/3 transcriptional activity. USP11 activity antagonized SMAD7 pathway inhibition, therefore SMAD7 was an unlikely USP11 substrate. Additionally, endogenous USP11 was not able to interact with any other SMADs besides SMAD7. Finally, SMAD7 is known for targeting the TGFβ R1 receptor (ALK5) for ubiquitylation by E3 ligases [7]. We therefore hypothesized that USP11 directed by SMAD7 plays a role in balancing receptor ubiquitylation.

### 3.5. USP11 interacts with ALK5

**Figure 4.**
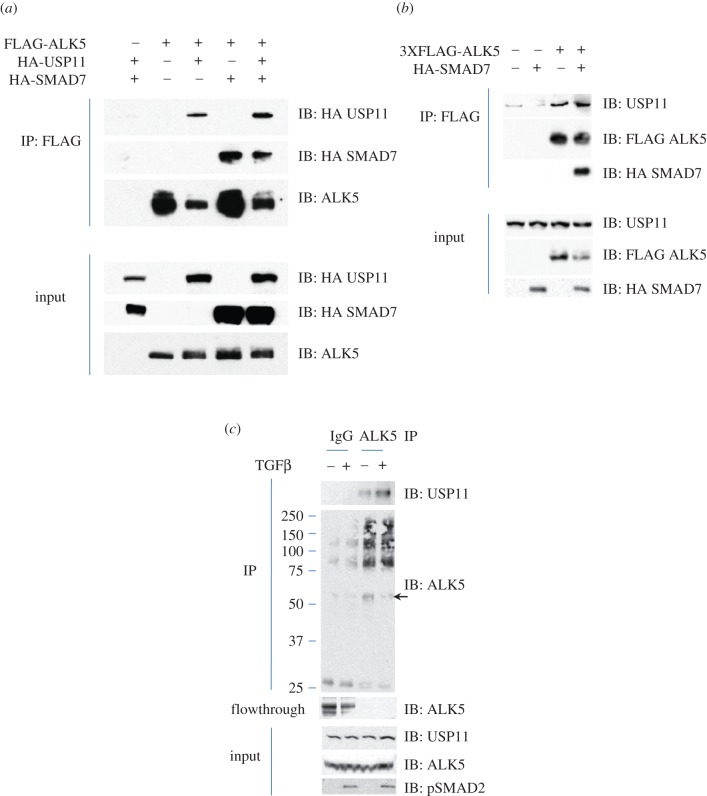
USP11 interacts with ALK5. (*a*) HEK293 cells were transiently transfected with FLAG-ALK5, HA-USP11 and/or HA-SMAD7, as indicated. Extracts or FLAG IPs were resolved by SDS–PAGE and immunoblotted with antibodies against HA-USP11, HA-SMAD7 and ALK5. (*b*) HEK293 cells were transiently transfected with 3XFLAG-ALK5, and HA-SMAD7, as indicated. Extracts or FLAG IPs were resolved by SDS–PAGE and immunoblotted with antibodies against endogenous USP11, HA-SMAD7 and 3XFLAG-ALK5. All immunoblots are representative of at least three biological replicates. (*c*) Lysates from HEK293 cells treated with vehicle or TGFβ (50 pM 45 min) were immunoprecipitated using pre-immune IgG or an ALK5 antibody covalently bound to Dynabeads. IPs, flow-through extracts and lysate inputs were immunoblotted with endogenous USP11, ALK5 and phospho-SMAD2 antibodies. The arrowhead denotes the native molecular weight ALK5.

HEK293 cells were transiently transfected with FLAG-ALK5 and HA-USP11 in the presence or absence of HA-SMAD7. FLAG-ALK5 interacted with HA-USP11, and this interaction was only slightly enhanced in the presence of over-expressed SMAD7 (figure 4*a*). Furthermore, FLAG-ALK5 was also able to immunoprecipitate endogenous USP11; however, over-expressing SMAD7 did not enhance the interaction between FLAG-ALK5 and endogenous USP11 (figure 4*b*). We also performed an endogenous ALK5 immunoprecipitation and were able to show endogenous USP11 in the ALK5 IPs. There did seem to be a slight enhancement of the interaction with TGFβ treatment (figure 4*c*). In ALK5 IPs, in addition to the predicted molecular weight bands, the ALK5 antibody also recognized high-molecular-weight cross-reacting bands. We therefore verified the loss of native molecular weight ALK5 in the flow-through extracts following ALK5 immunoprecipitation (figure 4*c*).

In light of the USP11–ALK5 interaction, we characterized the subcellular localization of endogenous SMAD7, USP11 and ALK5 to confirm that their interactions were not an artefact of the biochemical techniques used. We performed fixed-cell immunofluorescence on HaCaT keratinocyte cells and found USP11 to be both cytoplasmic and nuclear (see the electronic supplementary material, figure S5, top left panel). USP11 antibody specificity was confirmed using fixed-cell immunofluorescence in the presence or absence of USP11 knockdown using *siRNA* in two different cell lines (see the electronic supplementary material, figure S6). Consistent with previous reports, SMAD7 was observed mostly in the cytoplasm (see the electronic supplementary material, figure S5, middle left panel) [38,39]. Endogenous ALK5 was found mainly in the cytoplasm, as described in previous reports (see the electronic supplementary material, figure S5, bottom left panel) [40,41]. We demonstrated significant overlap between USP11 and SMAD7 in the cytoplasm (see the electronic supplementary material, figure S5, top right panel). We also demonstrate a considerable overlap of USP11 and ALK5 (see the electronic supplementary material, figure S5, middle right panel). As expected, ALK5 and SMAD7 overlap was seen in both the membrane and cytoplasm, consistent with reports of receptor internalization for both pathway signalling and receptor degradation [42].

### 3.6. USP11 deubiquitylates ALK5

**Figure 5.**
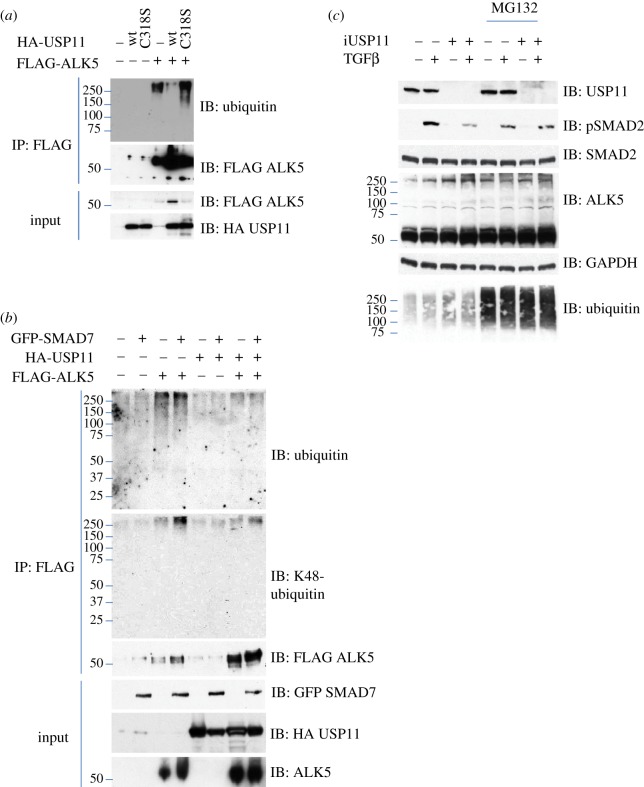
USP11 deubiquitylates ALK5. (*a*) HEK293 cells were trasfected with FLAG-ALK5 with or without a wt or catalytically inactive mutant (C318S) of HA-USP11. The FLAG-ALK5 IPs and extracts were resolved by SDS–PAGE and immunoblotted with antibodies against ubiquitin, FLAG-ALK5 and HA-USP11. (*b*) HEK293 cells were trasfected with FLAG-ALK5 with or without HA-USP11 or GFP-SMAD7, as indicated. The FLAG-ALK5 IPs and extracts were resolved by SDS–PAGE and immunoblotted with antibodies against ubiquitin, K48-linked polyubiquitin chain, FLAG-ALK5, GFP-SMAD7 and HA-USP11. (*c*) HEK293 cells were trasfected with FLAG-ALK5 with FoxO4 as control or USP11 si*RNA*, and treated with or without MG132 and/or TGFβ, as indicated. Extracts were resolved by SDS–PAGE and immunoblotted with antibodies against USP11, phospho-SMAD2, SMAD2, ALK5 and ubiquitin. All immunoblots are representative of at least three biological replicates.

Multiple TGFβ pathway members are ubiquitylated and could be potential deubiquitylation targets [11–13,17,18]. However, because USP11 interacts with ALK5 and positively regulates the TGFβ pathway dependent on its catalytic activity, ALK5 appeared to be a strong candidate for deubiquitylation by USP11. When over-expressed in HEK293 cells, FLAG-ALK5 is polyubiquitylated. Over-expressed wt USP11 was able to deubiquitylate ALK5. Catalytically inactive USP11 (C318S) could not (figure 5*a*), despite its ability to bind ALK5 (see the electronic supplementary material, figure S7). Over-expression of SMAD7 further increased ALK5 ubiquitylation, particularly K48-linked ubiquitin chains known to target proteins for proteasomal degradation. USP11 was able to reduce ALK5 polyubiquitylation, although not to basal levels. This denoted a receptor ubiquitylation balance between USP11 and SMAD7-bound E3 ligases (figure 5*b*). If USP11 does enhance pathway signalling through ALK5 deubiquitylation, then inhibiting the proteasome would negate any USP11 modulation. Consistent with this, we were able to show that the decrease in TGFβ-induced SMAD2 phosphorylation by USP11 depletion is abrogated by proteasomal inhibition (20 μM MG132, 3 h before TGFβ stimulation). We also see an increase in high-molecular-weight ALK5 bands both with USP11 depletion and MG132 treatment separately, but no further enhancement of these high-molecular-weight bands with MG132 and USP11 depletion together. As expected MG132 treatment increased general polyubiquitylation levels (figure 5*c*). This result indicates a central role for the proteasome in USP11 modulation of TGFβ pathway signalling.

### 3.7. USP11 knockdown inhibits epithelial to mesenchymal transition

**Figure 6.**
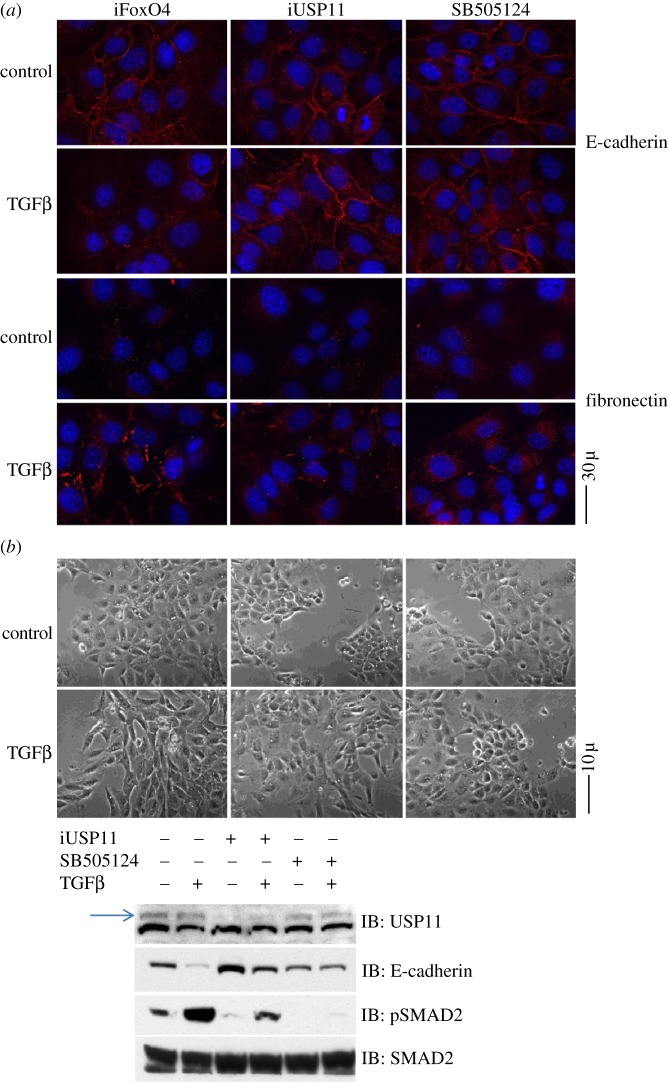
USP11 knockdown inhibits epithelial to mesenchymal transition. NMuMG cells were transiently transfected with *siRNA* targeting mouse FoxO4 as control or USP11 before being treated with 75 pM TGFβ for 24 h in the presence or absence of 1 μM TGFβ inhibitor SB505124. (*a*) E-cadherin and fibronectin immunofluorescence after TGFβ treatment (*b*) light microscopy of cells after TGFβ treatment. Western blotting of extracts from cells pictured were resolved on SDS–PAGE gels and blotted for USP11 (the arrowhead denotes USP11; a non-specific band appeared below USP11 in mouse cell extracts that was not present in human cell extracts), phospho-SMAD2, SMAD2 and E-cadherin.

Epithelial to mesenchymal transition (EMT) is a process, whereby epithelial cells undergo profound changes in shape and behaviour to become mesenchymal cells. EMT is a fundamental process during embryogenesis and organogenesis. The acquisition of the mesenchymal phenotype characterized by the loss of E-cadherin-mediated cell–cell adhesion and loss of apical basal cell polarity among others result in enhanced cellular plasticity. While the precise roles of EMT in cancer progression are still unclear, EMT may confer malignant traits such as motility, stemness, invasiveness and survival in cancer cells. EMT is also thought to play an important role in fibrosis. TGFβ is a potent inducer of EMT [43–45]. Given the impact of USP11 on the TGFβ pathway, we investigated whether USP11 was capable of altering TGFβ-induced EMT in NMuMG cells, a mouse mammary epithelial cell line. TGFβ treatment (75 pM for 24 h) of control FoxO4 *siRNA* transfected cells displayed a robust EMT response. Cells with *RNAi*-mediated depletion of USP11 showed a reduction in EMT induction after 24 h of 75 pM TGFβ stimulation, mimicking the effects of TGFβ inhibitor SB505124 (figure 6*a*,*b*) [46]. These effects were seen using immunofluorescence; E-cadherin, an epithelial marker, is clearly membranous in untreated control cells, while it disappears from the membrane with TGFβ treatment. Membrane E-cadherin persisted in both USP11-depleted and SB505124-treated cells despite TGFβ treatment. Fibronectin, a mesenchymal marker, was increased in the TGFβ-treated control cells as seen by immunofluorescence. In contrast, little or no increase was seen in the USP11-depleted and the SB505124-treated cells (figure 6*a*). Using phase contrast microscopy, it was found that TGFβ-treated control cells show a morphological shift from cuboidal (epithelial) to elongated (mesenchymal). SB505124-treated cells showed no morphological changes, while the USP11-depleted cells were a mix of mostly epithelial and some mesenchymal reflecting the transfection efficiency of USP11 *siRNA*. Western blotting of extracts of the same pictured cells shows a blunted TGFβ-induced reduction of E-cadherin upon USP11 depletion. Complete inhibition of the TGFβ pathway using 1 μM SB505124, added to cells 2 h prior to TGFβ treatment, had a similar but stronger effect (figure 6*b*). Clearly, *RNAi*-mediated USP11 depletion by multiple *siRNAs* in both mouse and human cells show the same inhibitory effects on TGFβ-induced phosphorylation of SMAD2 as well as EMT. This implies that these consequences are unlikely to be due to the off target effects of the *siRNAs* used and also highlights the global effects of USP11 across species.

## 4. Discussion

Despite a plethora of reports on TGFβ signalling regulation by E3-ubiquitin ligases, the DUBs that reverse or edit the effects of these E3-ubiquitin ligases have not received much scrutiny. To date, very few TGFβ pathway DUB regulators have been identified [11]. Here, we identify and characterize a new TGFβ pathway DUB: USP11.

We identified USP11 from a proteomic approach as an interactor of the inhibitory SMAD7 and further confirmed this interaction using a biochemical approach. Size-exclusion chromatography also alluded to the possibility of potential complex formation between USP11 and SMAD7. Despite its interaction with SMAD7, we found that USP11 enhanced TGFβ signalling and bound the TGFβ R1 receptor (ALK5). Pathway signalling was modulated through changes in SMAD2/3 phosphorylation. SMAD nuclear translocation and changes in transcriptional responses were also affected by USP11. Furthermore, we show that only wt USP11 is capable of eliciting and enhancing the R-SMAD transcriptional responses. By contrast, a catalytically inactive USP11 (C318S), still capable of binding ALK5, could not elicit the same response. Neither could the related DUB USP5. Therefore, the USP11 effects we observe on TGFβ signalling are specific and dependent on USP11 DUB activity. While USP15 was found as a potential SMAD7 interactor in our proteomic screens, subsequent biochemical approaches failed to show an interaction with any of the SMAD proteins.

We initially investigated SMAD7 as a potential target for USP11 deubiquitylation. However, USP11 enhancement of pathway signalling, among other results, was at odds with this target. ALK5 was the more logical target of USP11 deubiquitylation. USP11 was in fact capable of reducing receptor ubiquitylation when over-expressed, whereas the catalytically inactive USP11 was not. The reverse effects were seen with reduced USP11 expression using *RNAi*. The proteasomal inhibitor MG132 negated the effect of USP11 knockdown on both ubiquitylation and SMAD phosphorylation indicating a central role for proteasomal degradation in USP11–TGFβ pathway modulation.

**Figure 7.**
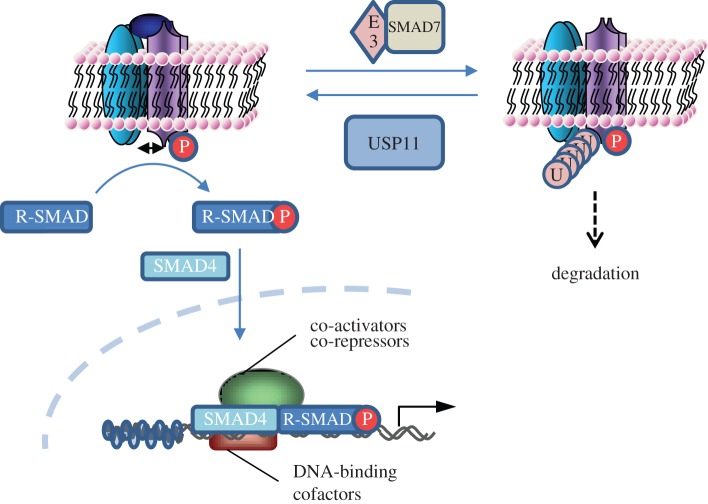
A schematic representation of the TGFβ pathway regulation by USP11. USP11 augments TGFβ signalling by deubiquitylating the type I TGFβ receptor, thereby counterbalancing the negative effect E3 ubiquitin ligases and SMAD7 have on the receptors.

SMAD7, a transcriptional target of TGFβ signalling, targets the active receptors for degradation by bringing E3 ligases (SMURF1/2 and WWP1) to ubiquitylate the receptor [17,18]. Depending on the relative activities of the E3 ligase and USP11, both bound to SMAD7, a balance between ubiquitylation and deubiquitylation could therefore decide receptor fate. A SMAD7-E3 complex targeting the receptor for ubiquitylation would lead to receptor degradation and signalling termination. Conversely, a SMAD7-USP11 complex would deubiquitylate the receptor, preventing its degradation and allow continued signalling (figure 7). This explanation would fit well with the experimental dataset we have achieved thus far. Furthermore, other BMP and TGFβ pathway receptors could also be involved in this ubiquitylation balance and therefore potential modulation targets for USP11.

A second mode of action that may not depend on the DUB activity of USP11, but its ability to bind and sequester SMAD7, could also be possible. This of course would also depend on the relative abundance of USP11 compared with SMAD7 and the stoichiometry of the interaction between them. However, this would be a complementary mechanism, because without its DUB activity, over-expressed USP11 (C318S) was not able to elicit or enhance a transcriptional response upon TGFβ stimulation. A third mode of action we feel is safe to disregard. USP11 may bind ALK5 and prevent the SMAD7-E3 ligase complex access to ubiquitylate the receptor. However, one would expect over-expression of the catalytically inactive USP11 that does bind to the receptor would provide such protection. Quite the opposite, it did not inhibit receptor ubiquitylation levels, they actually increased slightly (figure 5*a*). While the second mode of action needs to be further studied, it cannot fit within the scope of this paper. The study would need to take into account the intracellular movement of USP11 and SMAD7 and the stoichiometry of the interactions, both requiring extensive experimentation. Receptor movement and internalization should also be taken into account to complete the picture, as we cannot assume the receptor is stationary at the cell surface. While preliminary data for the second mechanism of action are promising, these have opened a much wider avenue of study than we intended to address with this paper.

Finally, we investigated whether USP11 was capable of modulating a TGFβ-induced EMT. EMT is a core feature of TGFβ pathway functions both in embryonic development and tumorigenesis. We found reduction of USP11 expression inhibited the pathway similar to TGFβ pathway blockade using a small molecule inhibitor. Both were able to reduce the EMT. This was particularly exciting, as modulating USP11 itself may open new avenues of anti-cancer drug discovery. While we chose EMT as a physiological readout of pathway modulation, other TGFβ-dependent physiological effects would also need to be taken into account during drug discovery especially where the TGFβ pathway acts both as a tumour promoter and suppressor. Modulating USP11 function within the TGFβ pathway would therefore provide a two-way level of control depending on the cellular context. It should be cautioned that directly targeting USP11 would have consequences beyond the TGFβ pathway as it has multiple reported targets in other pathways. Therefore, drug discovery should be concentrated on interfering with adaptor proteins that direct USP11 to certain parts of the target pathway, such as SMAD7 reported here for the TGFβ pathway. One should also consider that USP11 itself may be further modulated, not only by its adaptor proteins, but by post-translational modifications further increasing the potential ways to interfere with unwanted pathway signalling.

## 5. Material and methods

### 5.1. Materials

Cell culture media and antibiotics were purchased from Gibco. Foetal bovine serum (FBS) was from Hyclone. Polyethyleneimine (PEI) was from Polysciences (no. 24765). Lipofectamine 2000 was from Invitrogen (no. 52887). Transfectin was from Bio-Rad (no. 170-3551). Recombinant human TGFβ1 was from R&D (no. 240-B-002). Complete protease inhibitor cocktail-EDTA free was from Roche (no. 1187380001). RNA extraction kit was from Macherey-Nagel (no. 740955). Coomassie protein assay reagent was from Thermo Scientific (no. 1856209). Spin-X columns were from Costar (no. 8163). Chromatography columns were from Bio-Rad (no. 731-1550). GFP-Trap-A beads were from Chromatek (no. gta-20). Anti-FLAG M2 affinity gel was from Sigma (A2220). Glutathione sepharose beads were from GE healthcare (no. 17-0756-05). Protein G Dynabeads were from Invitrogen (no. 100.04D). NuPAGE 10 per cent bis–tris gels were from Invitrogen. Acrylamide was from Flowgen Bioscience (no. H16984). BS^3^ cross-linking reagent was from Thermo Scientific (no. 21585). Colloidal blue staining kit was from Invitrogen (no. LC6025). Nitrocellulose membranes were from Whatman (no. 10401191). Enhanced chemiluminescence (ECL) reagents were from Thermo Scientific (no. 34080). Western blot stripping buffer was from Thermo Scientific (no. 46430). Labtek chamber slides were from Nalge Nunc Int. (no. 154941). Glass bottom dishes were from WillCo (GWSt-3522). Vectashield mounting solution with DAPI was from Vectorlabs (no. H-1500). Antibodies to detect USP11 and USP15 were generated by injecting full-length GST-USP11/15 into sheep and affinity purified. FLAG-HRP and fibronectin antibodies were from Sigma (nos A8592, F3648). HA-HRP antibody was from Roche (no. 12013819001). SMAD7 antibody was from R&D (no. MAB2029). TGFβR1 antibody was from Santa Cruz Biotechnology (no. sc-398). Phospho-SMAD2 Ser465/467 antibody, SMAD2/3, GAPDH, E-cadherin and Lamin A/C were from Cell Signaling Technology (nos 3101, 3102, 2118, 4065, 2032, respectively). Ubiquitin antibody was from Dako (no. Z0458). Phospho-SMAD3 Ser423/425 antibody was from Rockland Inc. (no. 601-401-919). Goat anti-rabbit, mouse and sheep HRP-conjugated antibodies were from Pierce (nos 31460, 31430, 31480), respectively. Alexa Fluor 488 anti-sheep, 594 anti-mouse and 647 anti-rabbit were from Invitrogen (nos A11015, A11005, A31573, respectively). Nuclear cytoplasmic extraction reagents were from Thermo Scientific (no. 7883). Dual luciferase reporter assay kit was from Promega (no. E1960). RNA extraction kit was from Qiagen (no. 74004). iScript cDNA synthesis kit was from Bio-Rad (no. 170-8891). 2X SYBR Green Master was from Quanta Biosciences (no. 95071).

### 5.2. Plasmids

Mammalian expression constructs expressing human USP11, ALK5, SMAD1, 2, 3, 4 and 7 were cloned into pCMV5 or pCDNA-Frt-TO (Invitrogen) vectors with N-terminal FLAG, HA or GFP-tags. pCDNA-Frt-TO plasmids were used to generate stable HEK293 cell lines following manufacturers' protocol (Invitrogen). pGL4.11 LUC2p-SRE (SMAD-response element) reporter constructs were generated based on four repeats of the Smad-binding element (GTCTAG(N)C), as described previously [35,36]. Renilla-luciferase reporter was used as transfection control. All DNA constructs used were verified by DNA sequencing, performed by DNA Sequencing Service (MRCPPU, College of Life Sciences, University of Dundee, Scotland, www.dnaseq.co.uk) using Applied Biosystems Big-Dye Ver 3.1 chemistry on an Applied Biosystems model 3730 automated capillary DNA sequencer.

### 5.3. Cell culture, transfection and lysis

Cells were propagated in DMEM supplemented with 10 per cent FBS, 1 per cent penicillin/streptomycin and 2 mM l-glutamine. A 5 μg ml^−1^ insulin was added to the above media when propagating NMuMG cells. Cells were kept at 37°C in a humidified incubator with 5 per cent CO_2_. Cell lines stably expressing tetracycline-inducible GFP-USP11 were grown in media that additionally contained 100 μg ml^−1^ hygromycin and 15 μg ml^−1^ blasticidin. Human embryonic kidney (HEK293) cells were transfected using PEI, as described previously [47]. Human keratinocyte (HaCaT) and HEK293 cells were transfected using Lipofectamine 2000 or Transfectin according to manufacturers' protocol. Cells were seeded and transfected at 60 per cent confluency. They were allowed 48 h of growth in full growth medium before being treated with appropriate ligands and harvested. For protein applications, cells were scraped directly into cell lysis buffer (50 mM Tris–HCl pH 7.5, 1 mM EGTA, 1 mM EDTA, 1% Triton X-100, 1 mM activated sodium orthovanadate, 50 mM sodium fluoride, 5 mM sodium pyrophosphate, 0.27 M sucrose, 5 mM β-glycerophosphate, 0.1% β-mercaptoethanol and one tablet of protease inhibitor cocktail per 25 ml) and snap-frozen in liquid nitrogen. For RNA applications, cells were processed using an RNA extraction kit (Qiagen) according to manufacturers’ instructions. For luciferase assays (Promega), cells were prepared according to the manufacturers' protocol and assayed on a MicroLumat plus LB 96V luminometer from Berthold technologies.

### 5.4. Mass-spectrometric analysis

Mass-spectrometric analysis was performed by LC–MS–MS using a linear ion trap-orbitrap hybrid mass spectrometer (LTQ-Orbitrap, Thermo Fisher Scientific) equipped with a nanoelectrospray ion source (Thermo) and coupled to a Proxeon EASY-nLC system. Peptides were typically injected onto a Dionex Acclaim PepMap100 reverse phase C18 3 μm column, 75 μm × 15 cm (no. 160321), with a flow of 300 nl min^−1^ and eluted with a 40 min linear gradient of 95 per cent solvent A (2% acetonitrile, 0.1% formic acid in H_2_O) to 50 per cent solvent B (90% acetonitrile, 0.08% formic acid in H_2_O). The instrument was operated with the ‘lock mass’ option to improve the mass accuracy of precursor ions and data were acquired in the data-dependent mode, automatically switching between MS and MS–MS acquisition. Full scan spectra (*m*/*z* 340–1800) were acquired in the orbitrap with resolution *R* = 60 000 at *m*/*z* 400 (after accumulation to a target value of 1 000 000). The five most intense ions, above a specified minimum signal threshold of 20 000, based upon a low resolution (*R* = 15 000) preview of the survey scan, were fragmented by collision-induced dissociation and recorded in the linear ion trap (target value of 30 000). Data were analysed by searching the SwissProt/Human database using the Mascot search algorithm (http://www.matrixscience.com).

### 5.5. Immunoprecipitation

Snap-frozen cells were allowed to thaw on ice and centrifuged at 17 900*g* for 10 min at 4°C. Protein concentration was determined spectrophotometrically. Lysates (500 μg) were then immunoprecipitated using 10 μl packed beads (GFP-Trap, or FLAG) rotating for 2 h at 4°C. Protein-bound beads were then washed twice in lysis buffer with 0.5 M NaCl, and twice in buffer A (50 mM Tris–HCl pH 7.5, 0.1 mM EGTA, 0.1% β-mercaptoethanol) at 4°C. Samples were then reduced in 1× sample buffer (50 mM Tris–HCl pH 6.8, 2% SDS, 10% glycerol, 0.02% bromophenol blue, 0.1% β-mercaptoethanol), boiled at 95°C for 5 min prior to resolving by SDS–PAGE. For endogenous IPs, 5 mg of protein lysates was immunoprecipitated with 50 μl protein G dynabeads covalently bound to 5 μg SMAD7 or ALK5 antibody for 30 min at room temperature. Covalent binding was performed by using BS^3^ cross-linking reagent following manufacturers' protocol. Protein-bound beads were then washed thrice in phosphate-buffered saline (PBS), resuspended in 100 μl of PBS and transferred to a new tube. PBS was replaced with 50 μl 1× NuPAGE LDS buffer with 1 per cent β-mercaptoethanol. Samples were heated for 10 min at 70°C to elute the proteins prior to resolving by SDS–PAGE.

### 5.6. Gel filtration chromatography

HaCaT cells were lysed and filtered through Spin-X columns. A 1 mg of cleared protein extract was subjected to separation through a Superose 6 10/300 GL Column (GE Health Care), which was equilibrated, as described previously [48]. Eluting fractions (32 × 0.5 ml) were collected and processed for SDS–PAGE, as described earlier.

### 5.7. Immunoblotting

Snap-frozen cells were allowed to thaw on ice and centrifuged at 17 900*g* for 10 min at 4°C. Protein concentration was determined spectrophotometrically. Lysates (25 μg) or IPs were reduced in sample buffer and separated using 10 per cent denaturing gels and transferred onto a nitrocellulose membrane. Membranes were blocked with 5 per cent non-fat dry milk in Tris-buffered saline (50 mM Tris, 150 mM NaCl) containing 0.2 per cent Tween-20 (TBST), incubated overnight at 4°C with primary antibody, followed by incubation with horseradish peroxidase (HRP)-conjugated secondary antibody (1 : 5000). Detection was performed using ECL reagents.

### 5.8. Immunofluorescence

Cells seeded on Labtek chamber slides for fixed-cell immunofluoresence were allowed to grow for 24 h. Cells were fixed in 4 per cent paraformaldehyde for 20 min and permeabilized with 0.2 per cent Triton-X100 in PBS for 10 min at room temperature. Permeabilized cells were then incubated for 1 h at room temperature with blocking solution (5% donkey serum in PBS). Primary antibodies were added in blocking solution and incubated overnight at 4°C. Secondary fluor-conjugated antibodies were added after multiple washes in PBS for 90 min in the dark at room temperature. Alexa Fluor 488 nm anti-sheep (green), 594 nm anti-mouse (red) and 647 nm anti-rabbit (far red) were used. Vectashield mounting medium with DAPI was then used. Images were analysed using a Deltavision core restoration microscope (Applied Precision, USA).

### 5.9. *RNAi* and quantitative PCR

The siRNA and qPCR primer sequences used in this study are as follows:

Human *siRNAs* against USP11: *iUSP11-1* (5′–3′): GAUUCUAUUGGCCUAGUAU; *iUSP11-2:* CAGAGAUGAAGAAGCGUUA; *iUSP11-3* GUCAUAGAGCUGCCCAACA were from Sigma. Smartpool *siRNA* GGGCAAAUCUCACACUGUU; GAACAAGGUUGGCCAUUU; GAUGAUAUCUUCGUCUAUG; GAGAAGCACUGGUAUAAGC was from Thermo.

Human *siRNAs* against USP15: *iUSP15-1* (5′–3′): CUCUUGAGAAUGUGCCGAU; *iUSP15-2:* CACAAUAGAUACAAUUGAA; *iUSP15–3* CACAUUGAUGGAAGGUCAA were from Sigma.

Human *esiRNA* USP11 target sequence:

GGCATCTC AGGGAGAGACTGCTAGAAG GAGATGATTATGTGCTGCTCCCA GCGCCCTGCTTGGAACTACATGGT CAGCTGGTATGGCTTAATGGATGGCCAG CCACCTATTGAGCGCAAGGTAATAGAACTTCCTGGCATTCGGAAGGTGGAAGTGTAC CCACTAGAGCTACTGCTCGTTCAGCACAGT GATATGGAAACAGCTCTCACCATTCAGTT TAGCTATACTGATTCTGTGGAACTAGTCTTGCAAACAGCTCGGGAGCAGTTTCTGGTA GAGCCTCAGGAAGACACGCGCCTCTGGACCAAGAACTCAGAGGGCTCTTTGGATCGACTGTGTAATACACAGATCACGCTGCTTGA TGCCTGCCTTGAGACTGGGCAGTTGGTCATCATGGAGACTCGAAACAAAGATGGCAC TTGGCC

Mouse *siRNAs* against USP11: CUGUGAUCGUGGACACUUU; CCUACUAUGGUCUGAUACU; CAAAUAUGAUCUCAUCGCA were from Sigma.

qPCR primers used were:

GAPDH (F,R) (ATCTTCTTTTGCGTCGCCAG, GCTGAGACACCATGGGGAA)

FoxO4 (F,R) (TTGGAGAACCTGGAGTATGTGACA, AAGCTTCCAGGCATGACTCAG)

GADD45B (F,R)(AGTCGGCCAAGTTGATGAAT, CCTCCTCCTCCTCGTCAAT)

PAI-1 (F,R) (AGCTCCTTGTACAGATGCCG, ACAACAGGAGGAGAAACCCA)

SMAD7 (F,R) (CTGTGCAAAGTGTTCAGGTG, TTGAGAAAATCCATCGGGTA)

USP11 (F,R) (GTGTTCAAGAACAAGGTTGG, CGATTAAGGTCCTCATGCAG).

qPCR reactions were performed in triplicate on an iQ5 PCR machine (Bio-Rad) and data analysed using Microsoft Excel.

### 5.10. Statistical analysis

All experiments have a minimum *n* = 3. Error bars represent the standard deviation. Statistical comparisons (*p*-values) were obtained from Wilcoxon rank-sum tests.

## Supplementary Material

Supplementary Figure Legends and Data
