# Supplementary material for: USP11 augments TGFβ signalling by deubiquitylating ALK5
Source: Open Biol. 2012 Jun;2(6):120063. doi: 10.1098/rsob.120063 (PMC3390794; doi:10.1098/rsob.120063)
Supplement: Supplementary Figure Legends and Data [file rsob120063-s1.pdf]

SUPPLEMENTARY FIGURE LEGENDS

Supplementary Figure 1. TGFβ does not enhance interaction between SMAD7 and USP11. HEK293 cells were transiently transfected with empty FLAG vector or FLAG-SMAD7 alone, starved for 4 hours and stimulated for 1 hour with 50 pM TGFβ. FLAG-immunoprecipitates or extracts were resolved by SDS-PAGE and immunoblotted with antibodies against FLAG-SMAD7 and endogenous USP11.

Supplementary Figure 2. USP15 does not bind SMADs. HEK293 cells were transiently transfected with FLAG-SMAD1, 3, 4, 7, HA-USP11, and HA-USP15. FLAG-immunoprecipitates or extracts were resolved by SDS-PAGE and immunoblotted with antibodies against FLAG-SMADs, HA-USP11, and HA-USP15.

Supplementary Figure 3. Expression of DUBs in cell extracts used for TGFβ transcriptional reporter assays. HEK293 cells transiently transfected with SRE-luciferase, renilla-luciferase, HA-USP11, HA-C318S USP11 (DD), HA-USP5 and stimulated for 6 hours with or without 50 pM TGFβ as indicated. Cell extracts were resolved by SDS-PAGE and immunoblotted with antibodies against HA-USP and SMAD2.

Supplementary Figure 4. *RNAi* depletion of USP11 does not affect USP15 levels and *vice versa*. HEK293 cells were transiently transfected with multiple *siRNAs* targeting FoxO4 control or USP11 or USP15 separately. Extracts were resolved by SDS-PAGE and immunoblotted with antibodies against endogenous USP11, USP15, and SMAD2

Supplementary Figure 5. Cellular localisation of endogenous SMAD7, USP11, and ALK5. Fixed cell immunofluorescence of USP11, SMAD7, and ALK5 in HaCaT cells. Alexa Fluor 488 nm anti-sheep (USP11-green), 594 nm anti-mouse (SMAD7-red), and 647 nm anti-rabbit (ALK5-far red) were used.

Supplementary Figure 6. Fixed cell immunofluorescence of USP11. HeLa and HaCaT cells were transiently transfected with *siRNA* targeting USP11 to confirm USP11 antibody specificity for immunofluorescence applications.

Supplementary Figure 7. Catalytically inactive USP11 binds ALK5. HEK293 cells were transiently transfected with 3XFLAG-ALK5 with or without wild type or a catalytically inactive mutant (C318S) of HA-USP11. FLAG-immunoprecipitates or extracts were resolved by SDS-PAGE and immunoblotted with antibodies against FLAG-ALK5 and HA-USP11.

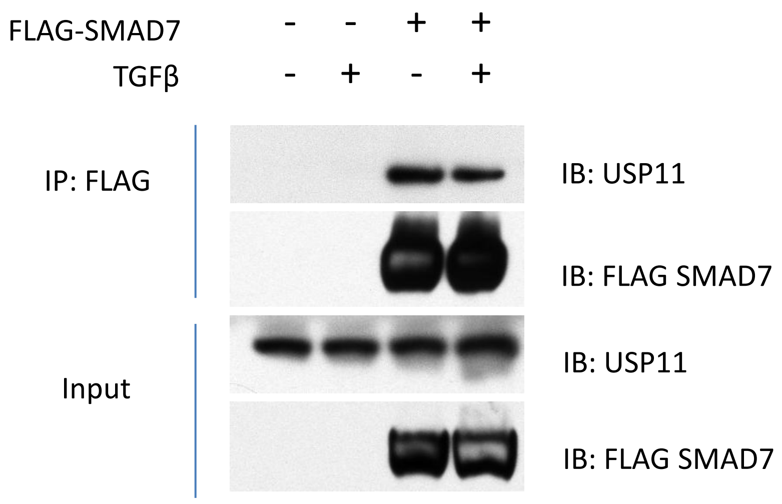

Al-Salihi *et al.* Supplementary Figure 1

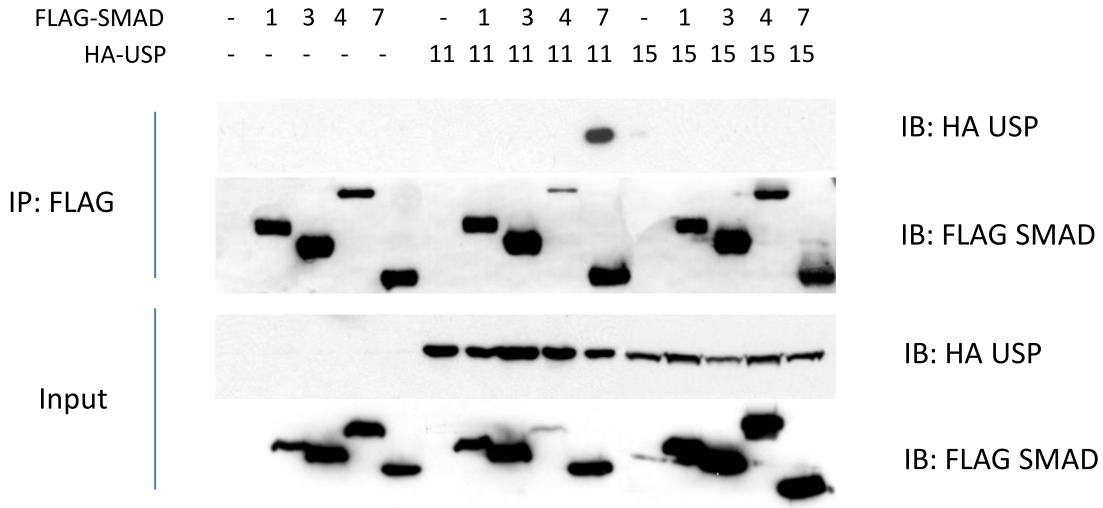

Al-Salihi *et al.* Supplementary Figure 2

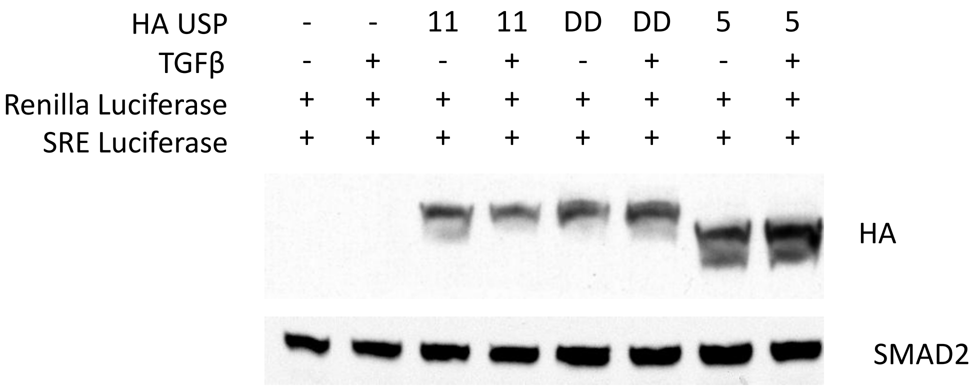

Al-Salihi *et al.* Supplementary Figure 3

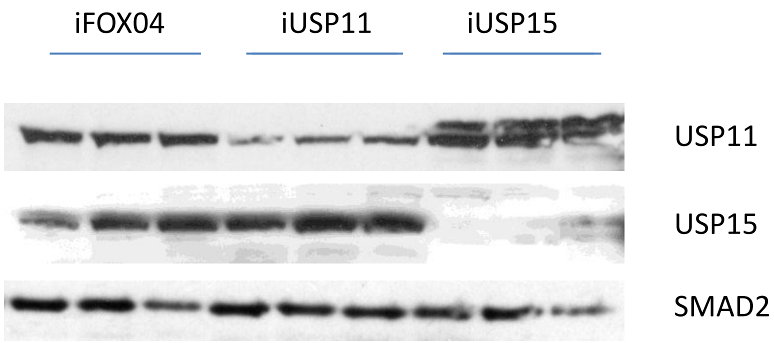

Al-Salihi *et al.* Supplementary Figure 4

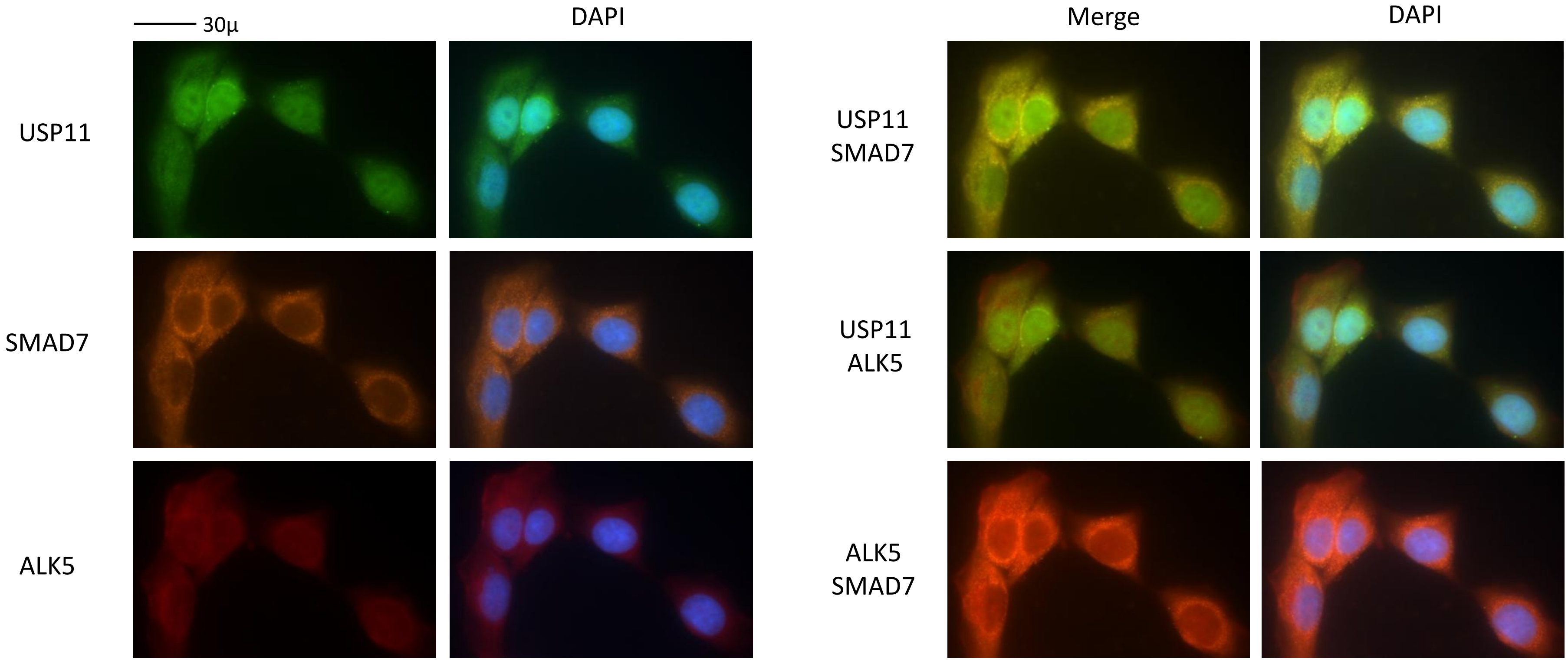

Al-Salihi *et al.* Supplementary Figure 5

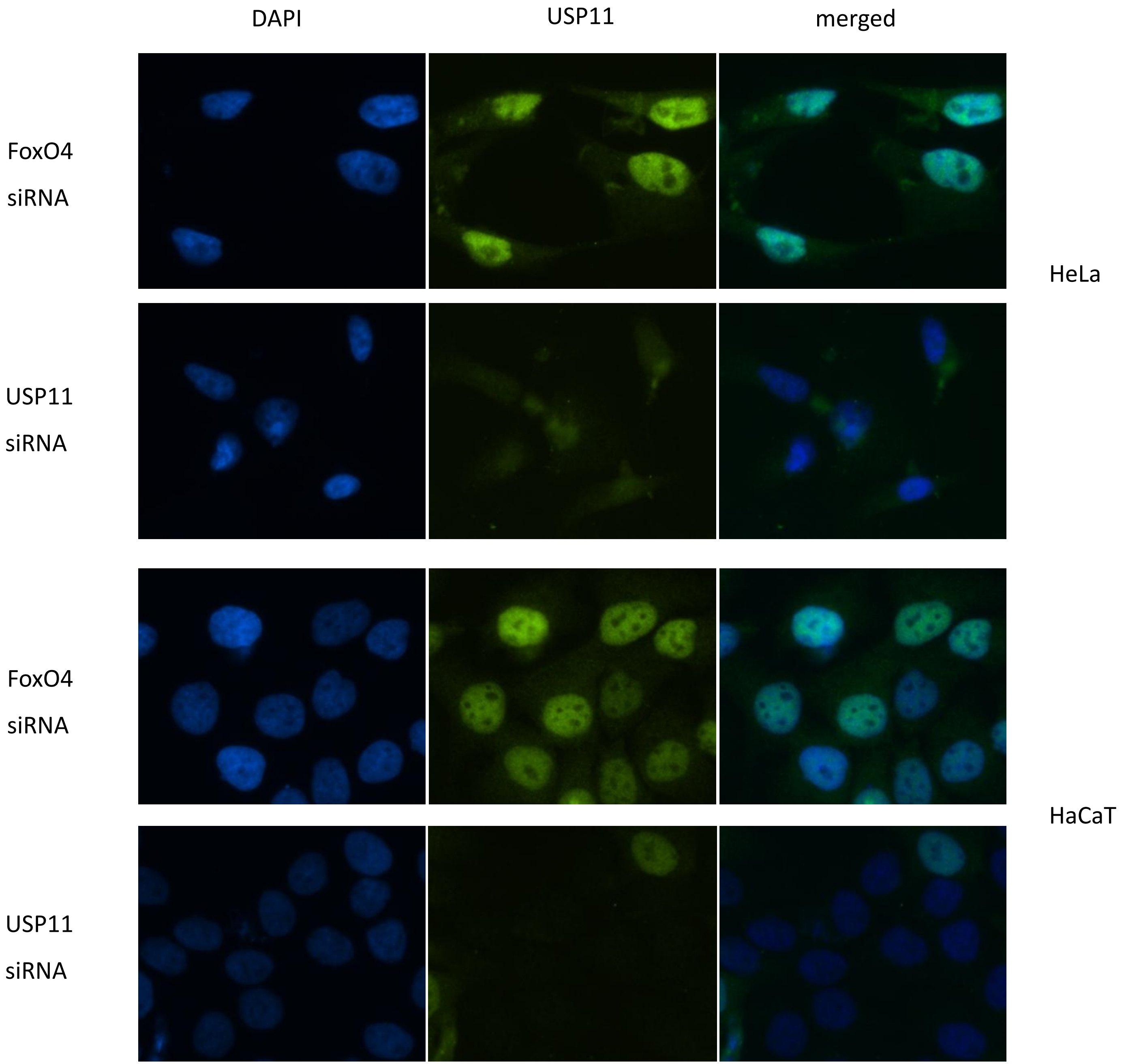

Al-Salihi *et al.* Supplementary Figure 6

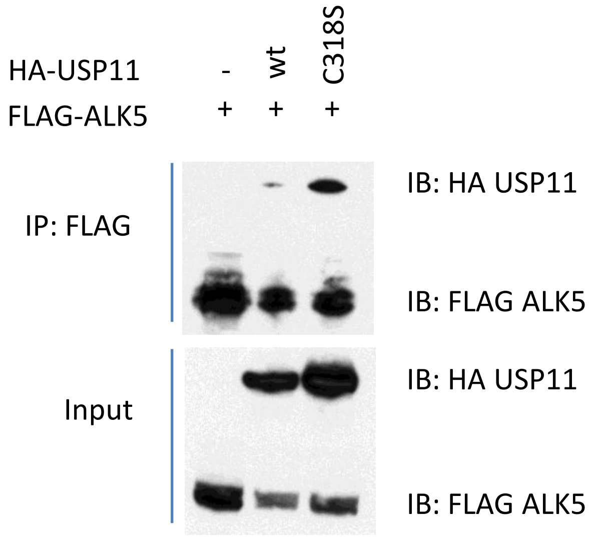

Al-Salihi *et al.* Supplementary Figure 7
